# Supplementary material for: JUND plays a genome-wide role in the quiescent to contractile switch in the pregnant human myometrium
Source: PLoS Genet. 2025 Jun 2;21(6):e1011261. doi: 10.1371/journal.pgen.1011261 (PMC12157839; doi:10.1371/journal.pgen.1011261)
Supplement: S1 Fig — Principal component analysis (PCA) of RNA-seq samples in non-laboring tissues (TNIL) and laboring tissues (TL). (PDF) [file pgen.1011261.s001.pdf]

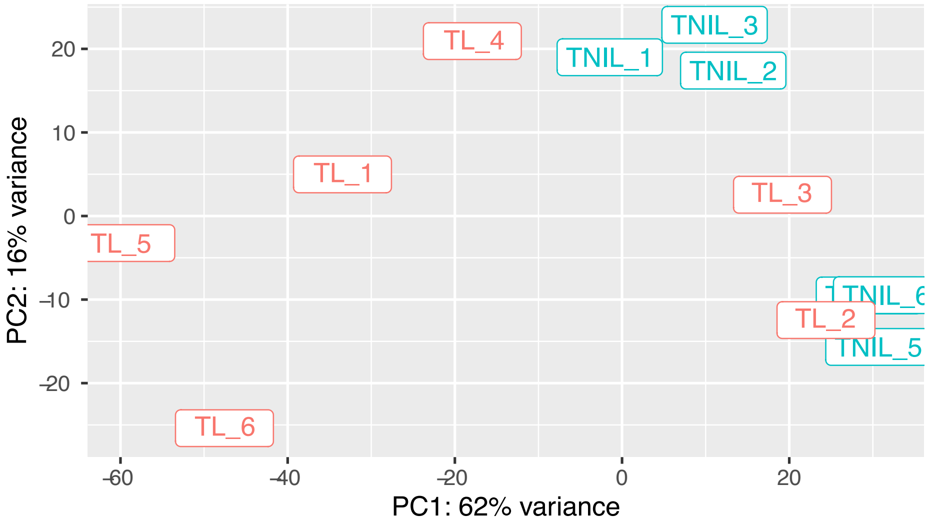

S1 Fig. RNA-seq samples cluster based on laboring status at the time of tissue collection. Principal component analysis (PCA) of RNA-seq samples in non-laboring tissues (TNIL) and laboring tissues (TL).
