# Supplementary material for: JUND plays a genome-wide role in the quiescent to contractile switch in the pregnant human myometrium
Source: PLoS Genet. 2025 Jun 2;21(6):e1011261. doi: 10.1371/journal.pgen.1011261 (PMC12157839; doi:10.1371/journal.pgen.1011261)
Supplement: S2 Fig — (A) Hierarchical clustering of JUND ChIP-seq samples from non-laboring (TNIL) and laboring (TL) tissues. Darker color indicates increased correlation. (B) Principal component analysis (PCA) of JUND ChIP-seq samples from non-laboring (TNIL) and laboring (TL) tissues. (PDF) [file pgen.1011261.s002.pdf]

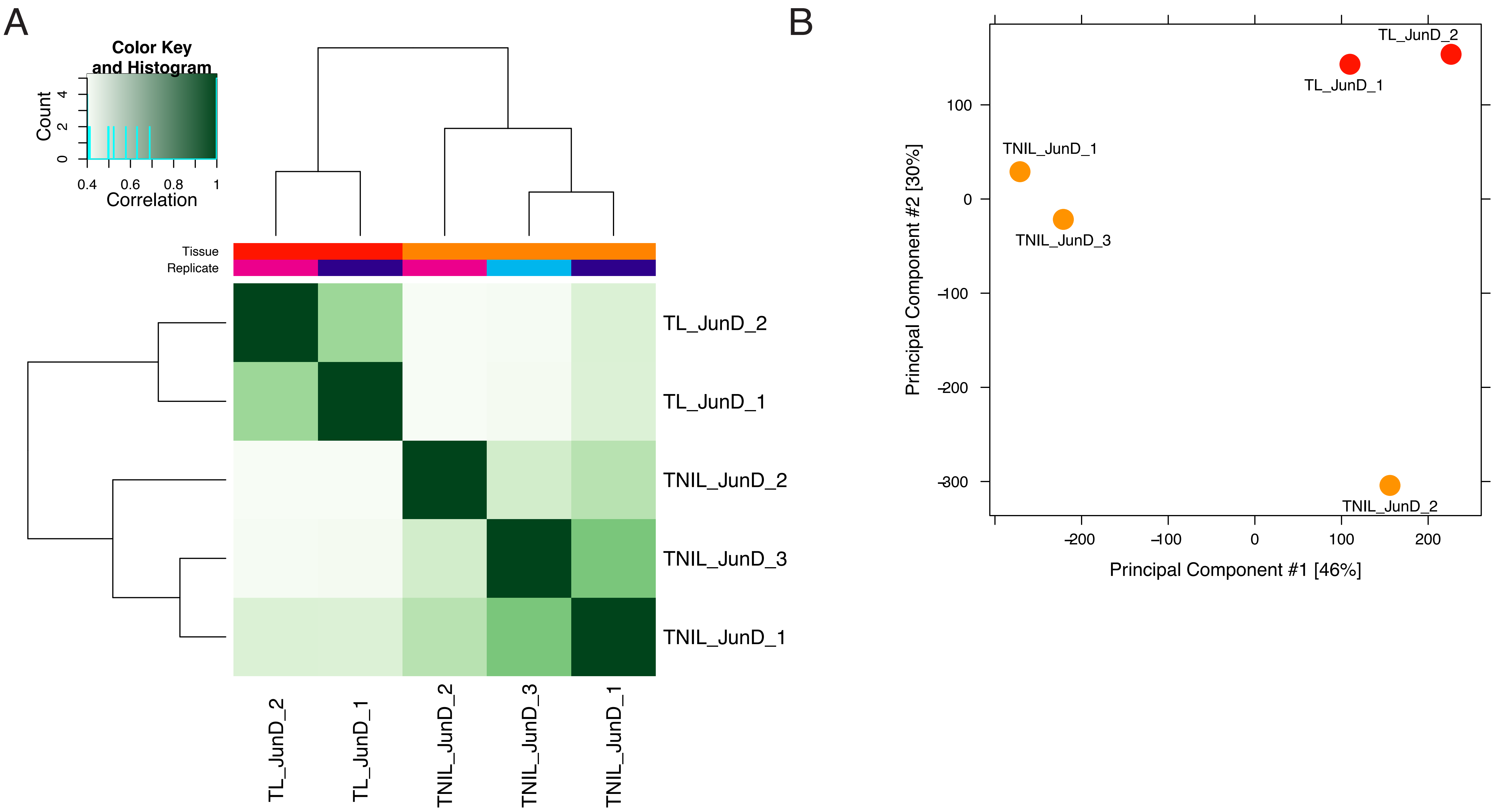

S2 Fig. JUND ChIP-seq samples cluster based on laboring status at the time of tissue collection.(A) Hierarchical clustering of JUND ChIP-seq samples from non-laboring (TNIL) and laboring (TL) tissues. Darker colour indicates increased correlation. (B) Principal component analysis (PCA) of JUND ChIP-seq samples from non-laboring (TNIL) and laboring (TL) tissues.
