# Supplementary material for: JUND plays a genome-wide role in the quiescent to contractile switch in the pregnant human myometrium
Source: PLoS Genet. 2025 Jun 2;21(6):e1011261. doi: 10.1371/journal.pgen.1011261 (PMC12157839; doi:10.1371/journal.pgen.1011261)
Supplement: Attachment — Submitted filename: JUND Paper revisions.docx [file pgen.1011261.s015.docx]

**Reviewers' comments:**

Reviewer's Responses to Questions

**Comments to the Authors:
Please note that one of the reviews is uploaded as an attachment.**

Reviewer #1: JUND plays a genome-wide role in the quiescent to contractile switch in the pregnant human myometrium
Khader et al., PLoS Genetics 2024

In the manuscript Khader et al, the non-laboring and laboring states of human myometrial samples was compared. They examined gene expression changes and the mechanisms driving these. They present a model in which JunD homodimers are bound at both enhancers and promoters in both non-laboring and laboring states with the Progesterone Receptor (PR) recruited by JunD at promoters. Their model proposes that an increase in Fos expression at the onset of labor results in JunD/Fos heterodimers enhancing gene expression.

Overall, this is an interesting paper and looks at an important biology system which is not highly studied. The quality of the data is high and overall the analysis is well done. This is relevant since the complexity of looking at human tissues can be confounding. The comparison of exon and intron data was excellent to drive the argument for transcriptional regulation rather than other aspects of gene regulation. Below are some comments:

1. The authors state at line 120 “Principal component analysis (PCA) of all samples revealed that replicates belonging to the same group cluster together and show similar expression trends (S1 Fig).”
I found this statement confusing since on the PCA, TL 2 and TL 3 seem to cluster with the TNIL. Some better description of the PCA would be more helpful. The samples are what they are, but does it suggest that TL is highly variable? It may also be that the diagnosis of active labor may be variable since the samples were collected by caesarean section.

**RESPONSE:** **Thank you for pointing this out. As you correctly note 2 of the TL samples are more similar to the TNIL group. We have revised this PCA statement; on line 138 of the changes tracked version the text now reads, “Principal component analysis (PCA) of all samples revealed that replicates belonging to the TNIL group clustered together. For the TL samples, 4 samples were clustered separately from the TNIL group, however, TL2 and 3 were clustered with the TNIL group, reflecting increased variability in the samples obtained in active labour (S1 Fig)”.**

2. Related to the above, gene expression analysis was done with the aggregated data. It would be useful to see the variability in the samples (could be box plots in supplemental?), for example, for Fos. If there is variability, is TL2 and TL3 outliers?

**RESPONSE: The heatmap shown in Figure 1 is based on the same RNA-seq samples as those shown in the PCA plot in SFig1 but focused on differentially expressed genes. This heatmap demonstrates that, although there is variability, all TL samples cluster together, suggesting consistent global gene expression patterns across the entire TL group for the genes identified as differentially expressed, including FOS.**

**Thank you for the suggestion of providing the individual read count data. Rather than doing this for a select few genes we have added read count data (PRM) for each replicate as a new tab in Table S1 and S3, providing both the exon and intron read counts. You can see that for FOS exon reads for example TL3 is low (113 RPM) compared to the TL average (504 RPM) whereas TL2 is very close to the average (435 RPM).**

3. The ChIP-seq data is very nice. Which samples were used for the ChIP-seq? Are they the same as the gene expression? Which ones?

**RESPONSE: We thank the reviewer for their positive assessment of our ChIP-seq data. Our initial plan was to use the TNIL and TL samples for both RNA-seq and ATAC-seq, however, the ATAC-seq we conducted failed the QC and was not high enough quality to sequence. As a result different samples were used for the JUND ChIP-seq as enough tissue was not available from the initial set used for RNA-seq.**

4. In the motif analysis, JunB keeps coming up as a candidate. While I understand that motif searches are not generally specific to a family member, it did make me wonder about other family members, particularly since JunB can function as a repressor. Is the expression of other Jun family members altered? While JunD has been explored before, a better justification for following up on JunD may help clarify the question.

**RESPONSE: Thank you for your insightful comment regarding the motif analysis and the role of other Jun family members, particularly JunB. As you correctly state, motif searches are often not specific to individual family members and indeed the JUND and JUNB motifs in the JASPAR database are virtually identical, see below. In fact the prevalence of the JUNB motif may be due to slightly less information content in this motif, T at position 11 weighted less than the corresponding T in the JUND motif.**

**JUND was chosen in particular as it shows increased protein expression in the laboring rodent and human myometrium (Nadeem et al., 2018). In addition, we were able to optimize an antibody for robust ChIP. JUND was also shown to be an activator of labor-associated genes (i.e. *Fos, Gja1*) (Mitchell et al., 2002, Shchuka et al., 2023) and more recently also explored for its role in activating *Ptgs2* (in association with FOSL2 and SOX4) as well as *Mmp11* (in association with FOSL2) (Khader et al., 2024).**

**The biological motivation for choosing JUND is given in the introduction at line 97 of the changes tracked version and we have added text to clarify the increase in protein levels; “In rodent and human tissues, the AP-1 family member, Jun proto-oncogene D (JUND) is expressed at increased levels, and displays increased protein accumulation in the nuclei of laboring myometrial cells compared to non-laboring pregnant tissues (Mitchell and Lye, 2002; Nadeem *et al.*, 2018a). Moreover, reporter assay experiments revealed enhanced expression from the *Gja1* promoter when members of the AP-1 subfamily FBJ osteosarcoma oncogene (FOS) and JUND were co-expressed in hamster myometrial cells (Mitchell and Lye, 2005).”**

JASPAR motifs:

JUNB:	MA0490.1

JUND: MA0491.1

Minor:

In line 522, the authors say for ChIP-seq they use “TNIL or TL (n = 2, each)”. However, in the figures, they have 3 and 2. In that regard, are these samples the same as the ones for the gene expression? If so, which ones were used?

**RESPONSE: No, the samples used for ChIP-seq are different from RNA-seq, as described above. We have fixed the error in the n values at line 601, the text now reads; “Briefly, 200 mg of flash frozen human myometrium tissues obtained from patients at TNIL (n=3) or TL (n = 2) were crushed and subjected to a secondary cross-linker”**

Figure S2, uses label LAB and TNIL rather than TL and TNIL. It would be good to be consistent.

**RESPONSE: Thank you for pointing out the inconsistency, the labels have been changed in Fig S2 for consistency.**

Reviewer #2: This is a strong study that addresses the epigenetic and transcriptomic transition experienced by the human myometrium as it transitions into labor. It performs a novel JUND ChIP-Seq analysis on clinical specimens and integrates this with an RNA-Seq analysis. Its main conclusion is that JUND shows dynamic binding to the genome during the TNIL to TL transition, with some loci gaining JUND binding, some losing JUND binding and some retaining similar levels of JUND binding. This latter group is interesting because these sites are also bound by the progesterone receptor (PR) in both TNIL and TL specimens. I have several requests:

1) The 88 genes that are upregulated across this data set plus the two other data sets referenced (Table S2) should be discussed further. Inspection of these genes reveals a large number of inflammation-associated genes, which is consistent with prior studies and becomes very clear when the list is sorted high to low, and this should be acknowledged. Importantly, many of these genes are highly specific neutrophil chemoattractants or neutrophil growth factors (CXCL1, CXCL2, CSF3) or neutrophil markers (CXCR2, CSF3R, S100A8, S100A9, IL1B). The detection of these genes is consistent with the known influx of neutrophils into the laboring myometrium, but the presence of these cells (and thus many gene products in the RNA-Seq data) should be mentioned as a limitation of the study. More generally, there should be discussions of the fact that the ChIP-Seq and RNA-Seq experiments were performed on mixtures of cell types, how this fact influences interpretations, and what cell type is the most prevalent and so primarily accounts for the observed changes. Are there any data on the myocyte:non-myocyte ratio in the term myometrium that would help tease this out? Could those loci with increased JUND binding at TL, being ones that are enriched for inflammatory targets, represent JUND binding in influxing or activated immune cells? This possibility is consistent with the fact that immune cells do not express PR and these regions do not show PR co-occupancy. Since presumably the authors do not intend to study neutrophils, the motif enrichment shown in Figure 1F should also be repeated without the genes known to be expressed relatively specifically by neutrophils. This might increase the significance of the enrichments detected. Published single cell RNA-Seq data set of the human myometrium could be used to parse out which genes are expressed by various kinds of immune cells more generally.

**RESPONSE: Thank you for raising this issue, we totally agree that bulk analysis is not ideal with complex tissues, and immune cells will be captured in our myometrium samples. Others have shown by histological analysis after a myometrium isolation technique similar to ours that the term non-labouring myometrium is composed of 65% smooth muscle, 33% ECM and 2% other cell types (vascular, neuronal, erythrocytes, immune cells; PMID: 23333540).**

**There are additional immune cells that infiltrate the myomrtrium at labour, however, the absolute numbers are still small compared to the smooth muscle cells. As you point out, the small percentage of immune cells can contribute to the RNA-seq results, however, should have less of an impact in the ChIP-seq as each cell has only 2 alleles that can be captured or not. Although some of the genes you mention are not known to be expressed in smooth muscle cells, some of them are expressed in smooth muscle cells and thought to have a role in supporting immune cell infiltration and function at labor.**

**Our published work in primary myometrial cells isolated from human myometrium (TL vs TNIL; PMID: 34526377) specifically excludes immune cells as these are not supported in culture, resulting in their removal during the culturing process. Multiplex protein analysis revealed that TL is associated with increased inflammation of the human uterus, as we detected significant differences in CXCL8, CCL2 and IL6 cytokines responsible for leukocyte migration and propagation of inflammation in the myometrium. Importantly, in this study CSF3, a cytokine regulator of myeloid progenitor cell proliferation and differentiation, exhibits the highest level of all proteins secreted by primary human myometrial cells (PMID: 34526377). We have additionally detected the expression of CSF3, CXCL1, CXCL2, and IL1B, in immortalized hTERT cells isolated from human myometrium and maintained in un-induced conditions (unpublished RNA-seq).**

**One of the triggers that elevates cytokines in the myometrium of healthy women during labour is a mechanical stretch of the uterine wall by the growing fetus at late gestation, which we demonstrated is an inducer of cytokine synthesis in human myometrial hTERT cells in culture (PMID: 24882387). We found that IL-6, CXCL8, CXCL1, MIF, and CSF3, were significantly increased by mechanical stretch. These stretch-induced cytokines are essential players during leukocyte (i.e., neutrophils) extravasation into the myometrium, they function as either pro-inflammatory agents (MIF, IL-6, CXCL1, CXCL8) or growth factors (CSF3) involved in neutrophils survival, proliferation, differentiation and function (PMID: 24882387). Among these cytokines, CXCL1 and CXCL8 had the highest stretch-induced increase in myometrial cells.**

**We have added to our manuscript to address these issues at line 153 of the changes tracked version the text now reads; “From our differential gene expression analysis, we found 88 labor-upregulated genes were amongst the 126 genes found in agreement between the other three datasets, which include known labour-associated genes, prostaglandin G/H synthase 2 (*PTGS2),* and FOS like 1 (*FOSL1*)(S2 Table)(Wu *et al.*, 1997; Mitchell and Lye, 2002; Mitchell *et al.*, 2004; Nadeem *et al.*, 2018). Also part of this list are several immune cell chemoattractants and growth factors. Specifically, interleukin 6 (*IL6*), chemokine ligand 2 (*CXCL2*), and colony stimulating factor 3 (*CSF3*), are produced by human myometrial cells in culture and are thought to have a role in supporting immune cell infiltration of the myometrium, an important signal for labour onset (Lee *et al.*, 2015; Shynlova *et al.*, 2021). S100 calcium binding protein A8 and 9 (*S100A8, S100A9*), however, are expressed in various immune cells at high levels (Austermann *et al.*, 2018) and may be present in this list due to the inclusion of immune cells in bulk tissue RNA-seq approaches.”**

2) It is unclear why PTGS2, IL6, CXCL2 and IL1B are singled out for special mention in the text and why certain genes are called out by name in Figure 1C.

**RESPONSE: PTGS2, IL6, CXCL2 and IL1B have all been previously identified as labour-associated genes in the myometrium, however, this sentence changed as detailed above to specifically highlight the genes involved in modulating immune cells. We have also added references to support our highlight of specific genes. In figure 1C we are not able to label all genes in a legible way and have therefore chosen a few which were of interest based on previous literature. Full tables of gene lists are provided in the supplement so that others can look for genes of interest.**

3) There are n=3 TNIL specimens but only n=2 TL specimens for the JUND ChIP-Seq. Can the authors comment upon whether this might have caused the skewing towards higher gene expression in the TNIL specimens?

**RESPONSE: We don’t think that the additional sample in the TNIL group contributed to the regions with increased binding at TNIL compared to TL. You can see from the heat map presented in figure 2C that there is good concordance between the TNIL replicates as well as the TL replicates at these regions. It is possible that removing one of the TNIL replicates would generate additional significantly different peaks in the diffBIND analysis as replicate TNIL JUND_2 is less similar to the other 2 replicates but this is unlikely to affect the 6,547 regions that we found were significantly different across all 5 samples, with increased binding at TNIL.**

4) Figure 1B, the lateral wisps of data points look artifactual (for the TNIL side of the plot, this is the contiguous “line” of data points that extend out to -7.5 log2fc and -7.5 -log10P; see attached screenshot). I wonder whether these peaks are small and so should be excluded? Is there anything special about them?

**RESPONSE: This is a known phenomenon with volcano plots, because the P values are calculated using chi square statistical analysis and this uses raw read count, low reads in one sample can cause this issue. The “wisps” reflect the mathematical relationship between fold change and p-values, which can be exposed when binding is low in one condition. For example, counts for two regions might be (5,5) vs (0,0) and (5,6) vs (0,1). These two regions would appear in the same "wisp". This is explained here in greater detail:** [**https://resources.qiagenbioinformatics.com/manuals/clcgenomicsworkbench/current/index.php?manual=Volcano_plots.html**](https://can01.safelinks.protection.outlook.com/?url=https%3A%2F%2Fresources.qiagenbioinformatics.com%2Fmanuals%2Fclcgenomicsworkbench%2Fcurrent%2Findex.php%3Fmanual%3DVolcano_plots.html&data=05%7C02%7Cja.mitchell%40utoronto.ca%7C06332a5495e84ebffd3a08dd626fc8d9%7C78aac2262f034b4d9037b46d56c55210%7C0%7C0%7C638774955633959112%7CUnknown%7CTWFpbGZsb3d8eyJFbXB0eU1hcGkiOnRydWUsIlYiOiIwLjAuMDAwMCIsIlAiOiJXaW4zMiIsIkFOIjoiTWFpbCIsIldUIjoyfQ%3D%3D%7C0%7C%7C%7C&sdata=x6PXy5p3QDEZ41XCFsXNc6w2e5vVuNeGLIw2zdI%2FtEM%3D&reserved=0)**. The combination of using a fold change and P value to highlight regions for further study helps to increase the focus on regions more likely to be involved in biological responses.**

5) The description of the JUND differential peak analysis is confusing (lines 185-195, Figures 2A and 2B). It starts off with what appears to be a tally of the absolute presence or absence of a peak, followed by a tally of peaks that show quantitatively different levels. If so, this should be made more explicit. But if I’m reading it correctly, I find this approach unnecessarily complex. How is it biologically meaningful to distinguish between whether a peak goes from present to “absent,” versus present to very low?

**RESPONSE: Sorry for this confusion, we have simplified this description considerably in the manuscript, see text below. Initially declaring the number of peaks identified in each set of samples gives the picture of how prevalent JUND binding is through the genome; a total of 76,987 peaks if all samples are considered. Simply identifying binding differences based on peak calls is not very stringent because it is actually not as simple as “absolute presence or absence of a peak”. Regions can be called as peaks in one set of samples and fall just below the threshold for peak calling in another set of samples whereas DiffBind analysis would not identify significant differences in this case. This is clear in the comparison between Fig2A and 2B; many of the TNIL only called peaks (36,642) do not meet the criteria for significance in the diffBind analysis (Fig2B) which only identified 6,547 peaks with differences based on our thresholds.**

**To clarify this point we have removed much of the text highlighting the differences based on peak overlaps. At line 224 of the changes tracked version the text now reads; “Our initial peak analysis identified 70,455 JUND peaks in the TNIL samples and 40,327 JUND peaks in the TL samples with 89% of the TL peaks overlapping a TNIL peak (Fig2A). 36,642 regions with peaks detected at TNIL did not display a significant peak at TL, suggesting a global loss of JUND binding occurs before labor onset (Fig 2A). To identify regions with significant differences in JUND association, differential peak analysis was conducted revealing a total of 6,740 peaks exhibiting significant differences between groups (log_2_ fold change ≥ |1|, *p*val < 0.01). 6,546 TNIL peaks, display a significant loss at TL (referred to as TNIL JUND peaks) and 192 peaks show a significant gain at TL (referred to as TL JUND peaks, Fig 2B, S5 Table).”**

Reviewer comment: Moreover, the key thing later on is to show that peaks with little change between TNIL and TL are the ones near PR-binding peaks. The authors should consider just dividing the peaks into three groups based upon cutoffs they choose for increasing and decreasing from TNIL to TL, and show a continuous plot in Figure 3C that demarcates where this cutoff is (and Figure 3C should be moved to Figure 2). This is the approach that was taken in the Dotts paper they cite, which I find more intuitive. Additionally, it’s confusing to claim that there is a “TNIL”-specific set of peaks when the top section of Figure 3C shows that many of these peaks are still there in the TL specimens.

**RESPONSE: Yes, we have divided the peaks into 3 groups based on the analysis in Figure 2B as stated in the text. This is the basis for the groups in Figure 3C. We included this 3C image in figure 3 for more clear comparison to figure 3D which displays the JUND, H3K27ac and PR enrichments for these 3 groups. We have adjusted the language throughout the text to refer to TNIL or TL enriched peaks rather than indicate that the peaks are “specific” to one labour phase. We hope this clarifies the data and the analysis.**

5) For Figure 2D, please perform a GO analysis of the shared peaks. This is particularly important since these regions are the ones co-occupied by PR.

**RESPONSE: We performed the GO analysis on the shared JUND PR promoters that were also labour upregulated genes, at line 364 the text states; “Gene ontology analysis revealed an enrichment in biological processes related to inflammation, cell communication, and regulation of cytokine production, essential processes for labor in the set of JUND/PR co-bound and upregulated genes (S8 Table).”**

6) Lines 203-207. I do not understand this sentence, and the lack of details in the S3 legend adds to my confusion. Are these just upregulated genes? Additionally, how can it be that S3 claims no difference whereas the similarly minor peaks differences in the shared categories of Fig 2D are discussed as though there were real?

**RESPONSE: The first sentence in this section, as well as figure S3, refers to JUND occupancy only at promoters of genes that are upregulated during labor whereas the rest of the analysis presented is on global peaks, promoter and distal. Since we later discuss the promoter association of JUND and PR we have removed this confusing sentence and figure S3. The rest of the section has been refined to more clearly describe that the gene profiles provided show examples of TNIL and TL enriched peaks.**

7) The choice to display data for ACTC1 and CCL24 in Figure 2E seems random and hence cherry-picked. Are these gene just examples of many that show the illustrated behavior? If so, what is the set of such genes and why not show more classic contraction associated genes instead of ACTC1?

**RESPONSE: These genes were chosen as examples as they were part of the GREAT enrichment set in each category and to highlight the TNIL or TL enriched JUND peaks.**

**We have now clearly indicated these are examples; at line 250 the text now reads, “Examples of specific genes linked to differentially bound JUND peaks are shown in Figure 2E. *ACTC1*, a cardiac muscle alpha actin encoding gene which is involved in muscle hypertrophy, and expressed at both TNIL and TL, displays increased JUND binding at TNIL (Fig 2E). At the *CCL24* gene, a labor-upregulated gene responsible for encoding a chemokine ligand that is involved in positive regulation of immune system processes, a TL enriched peak was observed (Fig 2E).”**

8) Although touched upon in the Dotts paper, which provided the PR ChIP-Seq data used here, the idea that PR binding would be associated with genes that are upregulated with the transition to labor is novel and contracts the prevailing idea in the field that labor onset in humans is associated with “functional progesterone withdrawal,” meaning that there is a loss of PR function, presumably associated with decreased PR binding to DNA, despite maintenance of high serum P4 levels. Not much in vivo data supports this idea however. So the present study’s description of continued PR binding to genes that are upregulated in the transition to labor (and very few genes with PR binding that are downregulated) is important, and I believe the paper would be enhanced if this was acknowledged and perhaps emphasized in its own right.

**RESPONSE: Thank you for suggesting we strengthen this finding. We initially did not want to focus as much on this as the PR data was previously published by Dotts et al. but to clarify this point we have made a number of changes.**

**In the introduction we have added; “Although progesterone bound to the PRB isoform of its receptor is generally thought to be involved in the repression of contractions during pregnancy, an interaction between the PRA isoform and AP-1 factors, in the absence of progesterone, was observed to cooperatively activate the expression of *Gja1* which is involved in myometrial contractions and labor timing (Döring *et al.*, 2006; Nadeem *et al.*, 2016).**

**In the results section we highlight that the PR ChIP will recognize both PRA and PRB; “Previous literature suggests a potential co-regulation of labor-driving genes, such as *GJA1*, by AP-1 factors and PRs, specifically the PRA isoform which is expressed at increased levels in the laboring myometrium (Nadeem et al., 2016). To explore this association on a broader scale, we analyzed available ChIP-seq datasets of the total PR cistrome (antibody does not distinguish PRA and PRB) and the histone mark H3K27ac, associated with active regulatory regions, from TNIL and TL human myometrium (Fig 3A)(Dotts *et al.*, 2022).”**

**In the discussion we have added the following; “Although the available PR ChIP-seq data does not distinguish between PR isoforms we propose that the activation of labour-associated genes bound by JUND and PR implicates unliganded PRA in this activating complex, similar to what was observed at the *Gja1* promoter (Nadeem et al., 2016). If true, this would further support the role for PRA and AP-1 factors as activators upon the functional withdrawal of progesterone in the human myometrium which is thought to be achieved by local progesterone metabolizing enzymes expressed in the human myometrium at increased levels during labor (Nadeem et al., 2016).”**

Minor:
1) The text in Figure S4A is unreadable. An excel file would be better.

**RESPONSE: This was already included as a supplemental table, We have included this files as supplemental table S6, we have removed Fig S4A and instead refer to the table.**

2) Figure S4B lacks an x-axis label. Also, I don’t understand why dots are plotted that seem to have Padj>0.05. It seems like some have Padj approaching 0.2 (Intracellular pH reduction), which means the enrichment is not significant and so should not be plotted. The same issue applies to Figure 5E.

**RESPONSE: We have instead included a table in the place of Fig S4B. This is S8 Table and the corresponding text at line 364 now reads; “Gene ontology analysis revealed an enrichment in biological processes related to inflammation, cell communication, and regulation of cytokine production, essential processes for labor in the set of JUND/PR co-bound and upregulated genes (S8 Table).”**

**For figure 5E we have modified the analysis slightly and therefore used a different visualization and GO tool, GREAT. Rather than mapping the eRNA regions to genes and TL expressed genes then conducting GO analysis on a smaller set of genes we have conducted the analysis on the eRNA regions using GREAT which maps regions to genes prior to GO analysis. This then aligns the analysis with that presented in figure 2. The text at line 413 now reads; “Further association of the regions with increased eRNA production to nearby genes identified enrichment in biological processes such as regulation of collagen production and genes linked to inflammatory phenotypes in mouse knockout studies (Fig 5E), as well as a significant enrichment of genes upregulated at TL (p < 3.756e-25, hypergeometric test).”**

3) Please clarify how Figure 1B differs from 1C. The text reads as though these are different data, not just different representations of the same data. Perhaps there is some subtlety regarding total transcripts and exon reads, but if so this should be made more clearly.

**RESPONSE: Figure 1B is a heat map representation which shows each of the replicates in the set of differentially expressed genes based on exon read counts. Figure 1C left shows the volcano plot based on exon reads and 1C right shows the volcano plot based on intron reads. The separate section that you are referring to, I think, is the description of intron-based gene expression analysis, Figure 1C on the right. We explain the intron analysis more extensively as it is less common and readers are therefore less familiar with it. We missed noting that we were referring to the right portion of figure 1C in the intron section and have now added that detail to the text.**

**At line 172 the text now reads; “Analysis of expression changes at the intron-read level uncovered a significant elevation of intronic reads at 661 genes, including *FOS* (Fig 1C, right, S3 Table), in the TL compared to the TNIL human myometrium, suggesting that the transition to labor onset is marked by an increase in gene transcription.”**

4) The redundant labeling of Fig 1B and 2C creates confusion. Why not just label the top of each heatmap with the orange and red bars, labeled respectively as TNIL and TL, and remove the orange/TNIL and red/TL legend labels on the side?

**RESPONSE: We have simplified the labelling as requested.**

5) What are the small histograms in the upper left of Fig 2C and S2A?

**RESPONSE: The histograms is a standard output from DiffBind, it shows how the color scale relates to read counts which were used to plot the heatmaps, this has been added to the figure legend.**

6) Line 151 – “74 genes” – should this be Supplemental Table 3 (not 2)? Additionally, I don’t see any list that has only 74 genes on it.

**RESPONSE: This data is in the second tab of table S2, intron comparison.**

7) The dot legend at the bottom of Fig. 1B has a grey category not present in the actual figure.

**RESPONSE: There are 3 grey colours in the volcano plot and these correspond to the 3 dots in the legend.**

8) The words “enriched” and “enrichment” are used in multiple different ways throughout the manuscript, and this reduces readability. Sometimes they refer to the statistical enrichments seen with a GO analysis, sometimes to the enrichment of binding at a particular locus seen with ChIP-Seq. I would suggest changing the second usage to peak “concentrations” or “intensities.” and “enrichment plots” to “peak profile plots”, to avoid confusion.

**RESPONSE: Thank you for this suggestion to increase the clarity of our work. We have changed the ChIP references to increased or decreased binding.**

9) The sentence in line 247-248 makes it sound like there are new data here that “confirm” the peak categories defined previously. But this was the analysis defined these categories in the first place, no? As above, I suggest moving Fig 3C to Figure 2 to improve clarity.

**RESPONSE: Our rationale for including 3C in the figure that compares JUND, PR and H3K27ac is for easy comparison to 3D and we feel it is important to keep this relationship in figure 3. The sentence referred to at 247-248 “Enrichment plots confirmed that JUND enrichment in TNIL peaks is largely lost in TL samples, with shared JUND peaks displaying JUND enrichment at both timepoints (Fig 3C).” was modified from the use of confirm to display as follows; “Enrichment plots display JUND enrichment at TNIL peaks is reduced or lost in TL samples, with shared JUND peaks displaying similar JUND enrichment at both timepoints (Fig 3C).” This sentence is merely intended to focus the reader back on the 3 groups before highlighting the comparison to PR and H3K27ac data.**

10) Line 249: “showed reduced absolute enrichment levels compared to TNIL ” – isn’t this observation that defined them as TNIL-specific peaks to begin with?

**RESPONSE: That is not what we intended to highlight. We were intending to point out that all the TL peaks are weaker than the peaks in the other groups; however, we have removed this sentence as it was somewhat redundant with the text pointing out the reduced overlap to H3K27ac regions.**

11) Figure S4B should be a main figure given its centrality to the main theme of the manuscript.

**RESPONSE: Thank you for this suggestion, however, we have replaced S4B with S8 Table since the table is an easier to evaluate representation of the results where P values and Padjusted values are easier to see, the table is also rather large and would not make a good main manuscript figure.**

12) Line 556: Please expound on the IDR approach to determining the combined peak set.

**RESPONSE: The Irreproducible Discovery Rate (IDR) is a statistical method for identifying reproducible and biologically relevant peaks in high-throughput sequencing experiments like ChIP-seq. It ranks peaks by signal strength across replicates, distinguishing true signals from noise using a bimodal model. Peaks with IDR ≤ 0.05 are considered significant, reducing false positives. IDR prioritizes rank consistency over genomic overlap, ensuring that peaks detected in both replicates with similar rankings are retained, while inconsistent peaks are filtered out. It does not strictly require peak overlap by a specific number of base pairs (e.g., 1 bp) but instead ranks peaks based on signal strength in each replicate and assesses their reproducibility. Peaks with similar rankings across replicates are considered reproducible. However, peaks that appear in only one replicate or have large rank differences are likely to be filtered out. IDR is a best practice for peak calling as recommended by ENCODE (PMID: 22955991).**

**A short description of these details has been added to the methods at line 635; “Significantly conserved peaks between biological replicates were combined using irreproducible discovery rate (IDR), a statistical method that ranks peaks by signal strength across replicates, using a bimodal model to remove less reproducible peaks (Li *et al.*, 2011).”**

Reviewer #3: In this study the authors identify differentially regulated genes expressed in human myometrial samples taken from term not in labor and term labor pregnancy. They also measure JunD, PR occupancy on promoters and correlated expression with transcription factor binding and identify JunD as a factor in changes in transcriptional pattern from TNIL to TL.

Sup Figure 1 line 119
The authors state “Principal component analysis (PCA) of all samples revealed that replicates belonging to the same group cluster together and show similar expression trends (S1 Fig).” Although the TNIL samples are clustered the TL samples are quite different and two are overlapping with the TNIL samples. This can also be seen in Fig 1b. Because of this I think that the TL samples can not be considered a cluster and the language used to describe them should be changed. Are there any clinically relevant differences between the TL samples that could be contributing to the variability?

**RESPONSE:** **Thank you for pointing this out. As you correctly note 2 of the TL samples are more similar to the TNIL group. We have revised this PCA statement; on line 138 of the changes tracked version the text now reads, “Principal component analysis (PCA) of all samples revealed that replicates belonging to the TNIL group clustered together. For the TL samples, 4 samples were clustered separately from the TNIL group, however, TL2 and 3 were clustered with the TNIL group, reflecting increased variability in the samples obtained in active labour (S1 Fig)”.**

Fig1F lines 150, 162.
In figure 1F the authors compare genes differently increased in TL based on intron reads to TL genes identified in other data sets. Since Exon reads were also measured within the same experiment as the Intron reads these two groups should be compared. They later state that this comparison was done and that AP-1 motifs were enriched but do not show the data.

**RESPONSE: We conducted a more global comparison of our data, both exon and intron, and other published data sets and arrived at the set of commonly upregulated genes. We have added the motif analysis for upregulated genes at the intron level to S4 table.**

**The text now reads, “In fact, when considering all 661 genes with increased intron levels in labor, AP-1 motifs remain significantly enriched in these gene promoters, along with NFKB (S4 Table).”**

Fig4 and 5
When comparing JunD peaks and PR and H3k27ac peaks why were only the shared TNIL and TL JunD peaks used in Fig 4 and 5? Based on the data from Fig 3 there are likely to be few hits, but they could be interesting and many reveal further patters that could better explain the regulation of expression of other genes such as CCL24 from Fig 2E. Also, does figure S4 include all JunD peaks or only the TNIL-TL shared peaks? Please clarify in the text.

**RESPONSE: Since the overlap between the TNIL/PR or TL/PR is so small we were not able to conduct any statistical analysis on these sets. For this reason we did not include the details. Figure S4 includes shared JUND peaks that are also bound by PR. The text now states; Using a hypergeometric analysis, we found a significant enrichment [p < 1.357e-32] of shared JUND and PR co-bound promoters in upregulated genes at labor (442 genes) and only a minimal overlap to downregulated genes (6 genes).**

Discussion and Results
The authors cite a mix of mouse and human data supporting their findings on the importance of PR in TL gene expression. In mice a decrease in progesterone occurs at the end of term and induces labor initiation. Since this does not occur in humans the authors should discuss what impact their findings have on understanding the differences in PR dynamics in human and mouse labor.

**RESPONSE: Thank you for pointing out that we should clarify that in humans there is a functional progesterone withdrawal that occurs in the myometrium. We have added this to the discussion also based on comments by reviewer 2 point 8; “ Although the available PR ChIP-seq data does not distinguish between PR isoforms we propose that the activation of labour-associated genes bound by JUND and PR implicates unliganded PRA in this activating complex, similar to what was observed at the *Gja1* promoter (Nadeem et al., 2016). If true, this would further support the role for PRA and AP-1 factors as activators upon the functional withdrawal of progesterone in the human myometrium which is thought to be achieved by local progesterone metabolizing enzymes expressed in the human myometrium at increased levels during labor (Nadeem et al., 2016).**

**Have all data underlying the figures and results presented in the manuscript been provided?**
Large-scale datasets should be made available via a public repository as described in the *PLOS Genetics* [data availability policy](http://track.editorialmanager.com/CL0/http:%2F%2Fjournals.plos.org%2Fplosgenetics%2Fs%2Fdata-availability/1/010f019532bb2718-08ca5e5f-ae1e-48d7-b1ec-008da23e2cbd-000000/QtFxfRCVVpE4OK5WMMenQSVjLK1h_Fe0SGW_LBssI0o=199), and numerical data that underlies graphs or summary statistics should be provided in spreadsheet form as supporting information.

Reviewer #1: Yes

Reviewer #2: Yes

Reviewer #3: Yes

PLOS authors have the option to publish the peer review history of their article ([what does this mean?](https://track.editorialmanager.com/CL0/https:%2F%2Fjournals.plos.org%2Fplosgenetics%2Fs%2Feditorial-and-peer-review-process%23loc-peer-review-history/1/010f019532bb2718-08ca5e5f-ae1e-48d7-b1ec-008da23e2cbd-000000/Edef_BdgmZsJvcuew7RBd-jrxTyQvpFOUpsBh8bKxOM=199)). If published, this will include your full peer review and any attached files.

If you choose “no”, your identity will remain anonymous but your review may still be made public.

**Do you want your identity to be public for this peer review?** For information about this choice, including consent withdrawal, please see our [Privacy Policy](https://track.editorialmanager.com/CL0/https:%2F%2Fwww.plos.org%2Fprivacy-policy/1/010f019532bb2718-08ca5e5f-ae1e-48d7-b1ec-008da23e2cbd-000000/ZhrQWdDVhWCsrvBB4pkKwYg6akiOSr2HPFqt49umbIs=199).

Reviewer #1: No

Reviewer #2: No

Reviewer #3: No

[NOTE: If reviewer comments were submitted as an attachment file, they will be attached to this email and accessible via the submission site. Please log into your account, locate the manuscript record, and check for the action link "View Attachments". If this link does not appear, there are no attachment files.]

**Figure resubmission:**

While revising your submission, please upload your figure files to the Preflight Analysis and Conversion Engine (PACE) digital diagnostic tool, <https://pacev2.apexcovantage.com/>. PACE helps ensure that figures meet PLOS requirements. To use PACE, you must first register as a user. Registration is free. Then, login and navigate to the UPLOAD tab, where you will find detailed instructions on how to use the tool. If you encounter any issues or have any questions when using PACE, please email PLOS at figures@plos.org. Please note that Supporting Information files do not need this step. If there are other versions of figure files still present in your submission file inventory at resubmission, please replace them with the PACE-processed versions.

**Reproducibility:**

To enhance the reproducibility of your results, we recommend that authors deposit laboratory protocols in [protocols.io](http://protocols.io/), where a protocol can be assigned its own identifier (DOI) such that it can be cited independently in the future. Additionally, PLOS ONE offers an option to publish peer-reviewed clinical study protocols. Read more information on sharing protocols at <https://plos.org/protocols?utm_medium=editorial-email&utm_source=authorletters&utm_campaign=protocols>
